# FMRFamide-Like Peptides (FLPs) Enhance Voltage-Gated Calcium Currents to Elicit Muscle Contraction in the Human Parasite *Schistosoma mansoni*

**DOI:** 10.1371/journal.pntd.0000790

**Published:** 2010-08-10

**Authors:** Ekaterina Novozhilova, Michael J. Kimber, Hai Qian, Paul McVeigh, Alan P. Robertson, Mostafa Zamanian, Aaron G. Maule, Tim A. Day

**Affiliations:** 1 Department of Biomedical Sciences and Neuroscience Program, Iowa State University, Ames, Iowa, United States of America; 2 School of Biological Sciences, Queen's University Belfast, Belfast, Northern Ireland, United Kingdom; Uniformed Services University, United States of America

## Abstract

Schistosomes are amongst the most important and neglected pathogens in the world, and schistosomiasis control relies almost exclusively on a single drug. The neuromuscular system of schistosomes is fertile ground for therapeutic intervention, yet the details of physiological events involved in neuromuscular function remain largely unknown. Short amidated neuropeptides, FMRFamide-like peptides (FLPs), are distributed abundantly throughout the nervous system of every flatworm examined and they produce potent myoexcitation. Our goal here was to determine the mechanism by which FLPs elicit contractions of schistosome muscle fibers. Contraction studies showed that the FLP Tyr-Ile-Arg-Phe-amide (YIRFamide) contracts the muscle fibers through a mechanism that requires Ca^2+^ influx through sarcolemmal voltage operated Ca^2+^ channels (VOCCs), as the contractions are inhibited by classical VOCC blockers nicardipine, verapamil and methoxyverapamil. Whole-cell patch-clamp experiments revealed that inward currents through VOCCs are significantly and reversibly enhanced by the application of 1 µM YIRFamide; the sustained inward currents were increased to 190% of controls and the peak currents were increased to 180%. In order to examine the biochemical link between the FLP receptor and the VOCCs, PKC inhibitors calphostin C, RO 31–8220 and chelerythrine were tested and all produced concentration dependent block of the contractions elicited by 1 µM YIRFamide. Taken together, the data show that FLPs elicit contractions by enhancing Ca^2+^ influx through VOCC currents using a PKC-dependent pathway.

## Author Summary

Schistosomiasis (bilharzia) is caused by infection with trematodes of the genus *Schistosoma*. The disease afflicts over 200 million people, with the bulk of the disease burden focused in some of the world's poorest countries. Schistosomiasis control rests largely on chemotherapy with a single drug, praziquantel, a precarious situation calling for the discovery and development of new antischistosomal agents. One hindrance to the discovery of new drugs is a deficiency of knowledge regarding some basic biological processes of these parasitic worms. Here, we take significant steps toward the elucidation of signaling and pathways involved in schistosome neuromuscular control, a central biological function with proven vulnerability to chemotherapeutic intervention. Neuropeptides are known to be important in flatworm muscle control and here we find that FMRFamide-like peptides act to contract schistosome muscle by enhancing calcium influx through voltage-operated calcium channels. We also found that the receptor for the myoexcitatory neuropeptides uses a protein kinase C pathway to stimulate the voltage-operated calcium channels. Understanding the molecules involved in the neuromuscular physiology of these worms helps to identify potentially useful targets for a new generation of antischistosomal drugs.

## Introduction

Schistosomes continue to inflict widespread suffering, infecting over 200 million worldwide, yet a proportional lack of scientific attention leaves schistosomiasis as one of the most prevalent neglected tropical diseases [1], [2]. Significant advances in schistosomiasis control have been achieved in the past few years, due primarily to well-organized control programmes that include aggressive chemotherapy [3], [4]. While these programmes produce unquestionable public health benefit, their assertive use of praziquantel raises concern about the potential emergence of resistance to the drug, which is the only antischistosomal available in most of the world. The schistosomiasis community has recognized the urgent need for new chemotherapeutic agents [5], [6].

Current approaches to drug discovery are significantly hindered by large gaps in our understanding of the basic biology of schistosomes at a molecular level. For example, even though most anthelmintic drugs have effects on the neuromuscular systems of worms, very little is known about the specifics of neuromuscular anatomy and physiology, nor the mechanisms involved in neuromuscular control.

The neuropeptidergic system in parasitic flatworms is known to be remarkably rich [7], [8]. Flatworm Phe-Met-Arg-Phe-amide (FMRFamide)-like peptides (FLPs), one particular group of Arg-Phe-amides (RFamides), are known to play a central role in parasite neuromuscular biology; they are widespread throughout the nervous system of every flatworm examined, and they produce potent excitation of flatworm muscle [9], [10], [11], [12], [13], [14]. The RFamides present in vertebrates are structurally dissimilar to those in helminths, suggesting that flatworm FLPs and associated signaling molecules represent opportune targets for therapeutic control. Our objective here was to discover the mechanism by which these FLPs induce contraction of schistosome muscle fibers.

## Materials and Methods

### Parasites and Muscle Fiber Dispersion

The studies here are focused on muscle fibers dispersed from adult *Schistosoma mansoni*. The fibers are obtained from adult worms recovered 45-60 days post-infection from the portal and mesenteric veins of female mice supplied by the Biomedical Research Institute, Rockville, MD USA [15]. Iowa State University's Institutional Animal Care and Use Committee (IACUC) approved the role of the mice.

The incubation medium used for the fibers was based on a modified Dulbecco's Modified Eagle's Medium (DMEM, Sigma-Aldrich, USA) which was diluted to 67% of normal concentration and to which were added the following: 2.2 mM CaCl_2_, 2.7 mM MgSO_4_, 0.04 mM Na_2_HPO_4_, 61 mM glucose, 1.0 mM dithiothreitol, 10 µM serotonin, 1% gentamicin solution (Roche Applied Science, USA), 15 mM 4-(2-hydroxyethyl)-1-piperazine ethanesulfonic acid (HEPES) (pH 7.4). The dispersion medium was this same medium to which was further added 1 mM ethyleneglycol-bis-(β-aminoethyl ether) N, N, N' N'-tetraacetic acid (EGTA), 1 mM ethylenediamine tetraacetic acid (EDTA), and 0.1% bovine serum albumin.

**Figure 1:**
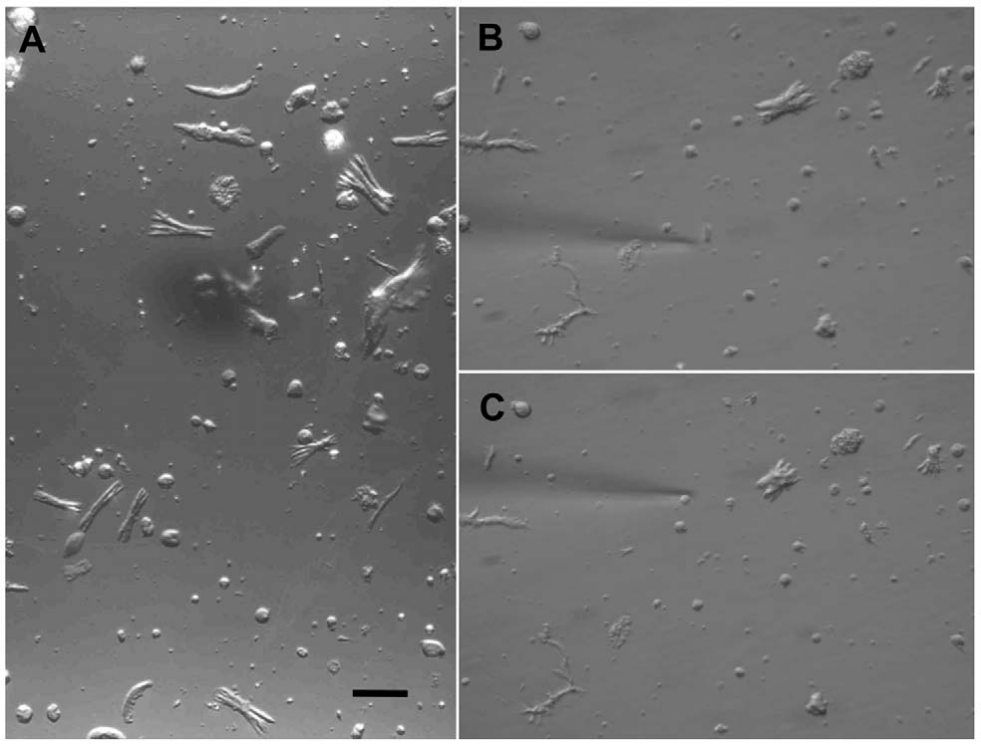
Contractile muscle fibers can be dispersed from adult schistosomes. (A) A typical field from a dish of the dispersion described in the methods. Amongst some debris and other cell types, there are a significant number of frayed muscle fibers which contract in response to the application of exogenous stimuli. (B) One specific frayed muscle fiber is approached by a pipet loaded with 1 µM YIRFamide dissolved into the same incubation medium in which the fiber is resting. (C) Pressure ejection of the YIRFamide onto the muscle fiber induces an abrupt contraction. For all three pictures the scale bar is 25 µm.

Briefly, live worms (25–30 pairs) were rinsed four times with the dispersion medium described above and then chopped approximately 35–40 times with a razor blade. Resultant worm pieces were rinsed four times, then incubated for 30 min at 37°C on a shaker table with the dispersion medium with added papain (1 mg/ml, Roche Applied Science, USA). After the enzymatic medium was removed, the worm pieces were rinsed twice with the enzyme free dispersion medium, then incubated for an additional 10 min. After a final rinse, worm pieces were mechanically disrupted by forcing them back and forth through the orifice of a Pasteur pipet, creating a suspension of some partially digested worm pieces and some dispersed cells and muscle fibers (Figure 1). This suspension was plated into plastic Petri dishes in the dispersion medium and was allowed to settle for one hour in the cooler at 19°C. During this hour of incubation, many dispersed muscle fibers partially adhered to the bottom of the Petri dishes. More detailed descriptions of these methods have been published [10], [16]. For both the contraction and the patch-clamp experiments, the Petri dishes were warmed to 35°C for 15–30 min prior to experimentation, and the experiments were performed in room air with the temperature held at 35°C via a culture dish heater and controller (DH-35 and TC-324B, Warner Instruments, USA).

### Contraction Studies

The contraction studies utilize these dispersed muscle fibers in the DMEM-based incubation medium described above. Quiescent fibers bathing in the incubation medium are individually perifused with test media in order to examine the ability of those media to elicit contraction. In a few instances, the incubation medium is substituted for an “inorganic” version, primarily in an effort to control precipitating interactions between various test agents and the phosphates in the incubation medium.

Observations of individual muscle fiber contractions were carried out using an inverted microscope (Nikon Eclipse TS 100) attached to camera, video-recorder and a monitor. These test media were pressure ejected through a pulled glass pipet onto individual quiescent muscle fibers, and a fiber was considered to have contracted if it shortened to less than 50% of its pretreatment length in 20 s. These contractions are very rarely equivocal. For each plate, 25–40 fibers were tested and the percentage of fibers contracting for that plate represents n = 1. Each data point is given as the mean ± SEM for at least five plates tested on cell preparations done on at least three different days.

Three different test media were applied to elicit contractions in these experiments.

1. The flatworm-derived neuropeptide Tyr-Ile-Arg-Phe-amide (YIRFamide). Amongst the earliest discovered flatworm neuropeptides was YIRFamide from *Bdelloura candida*, and it was soon thereafter found to have potent myoexcitatory action on a number of flatworm preparations including schistosomes [9], [10], [11], [12], [14], [17]. Similar neuropeptides (e.g. GYIRFamide, RYIRFamide and YMRFamide) have also been discovered in the phylum [8]. YIRFamide (Invitrogen, USA) was dissolved in the standard incubation medium at 1 µM.
2. A depolarizing extracellular medium with elevated [K^+^]. The depolarizing medium is identical to the standard incubation medium, except that the [K^+^] is raised from 5.4 mM to 25 mM, and the [K^+^] x [Cl^−^] product is kept constant by substitution of some of the NaCl with Na-gluconate.
3. Caffeine. Caffeine was dissolved at 1 mM in the standard incubation medium [18].

Agents tested for their ability to block contractions were introduced into the incubation medium 15 min prior to testing with the contractile agents. Stock solutions for the water-soluble test agents (10^−2^ M) were prepared in water. Stock solutions for the remaining blockers were prepared at the same concentration in DMSO, with the exception of nicardipine, which was dissolved in absolute ethanol. Due to light activation of calphostin C, stock solutions and dilutions were made in dim light and protected from light by wrapping containers in aluminum foil. All of these stock solutions were further diluted in the incubation medium.

### Patch-Clamp Experiments

The standard extracellular bathing solution for electrophysiological recordings was (mM): NaCl 130; KCl 5.0; CaCl_2_ 2.0; glucose 10.0; MgCl_2_ 1.0; HEPES 20.0 (pH 7.3). The incubation medium in which the dispersed fibers were resting was exchanged for this standard extracellular bathing solution about 10 min before recording. A fiber targeted for recording inward currents was then locally perifused with an extracellular recording solution with elevated Ca^2+^ and Ba^2+^ in order to maximize the inward currents, composed of (mM): HEPES 20.0; MgCl_2_ 1.0; glucose 10.0; NaCl 112.0; KCl 5.0; CaCl_2_ 2.0; BaCl_2_ 15.0 (pH 7.3). The electrode solution contained (mM): HEPES 10.0; MgCl_2_ 2.0; glucose 10.0; CsMethaneSulfonate 130.0; CsCl 20.0; EGTA 10.0 (pH 7.3). All solutions used for electrophysiology were filtered at 0.2 µm.

The application of the extracellular recording solution and the drug-containing extracellular solutions utilized a SF-77B Perfusion Fast-Step (Warner Instrument Corporation, USA) apparatus for the rapid changes from control to drug-containing solutions and subsequent return to control solution for washout. Syringes with solutions were mounted approximately 14 inches over the preparation to produce a gravity-fed flow rate of approximately 0.5 ml/min.

Whole-cell recordings were performed using Axopatch 200B (Axon Instruments, USA) patch clamp amplifier. Experiments were controlled and data recorded using pCLAMP software (pCLAMP 8.0) and the Digidata 1320 analog-to-digital interface (Axon Instruments, USA). Currents were amplified, low-pass filtered at 1 kHz, digitized at 11.1 kHz and stored on a personal computer.

Patch-clamp pipets were double-pulled from borosilicate glass capillaries (outer diameter 1.2 mm, inner diameter 0.68 mm; World Precision Instruments, USA) using the PC-10 Micropipette Puller (Narishige, Japan). Since isolated S. *mansoni* fibers are relatively small (15–20 µm), successful seals and whole-cell configuration were obtained using patch electrodes with resistances that usually ranged from 15 to 20 MΩ. Because the voltage operated inward currents from the dispersed *S. mansoni* muscle fibers are also relatively small (less than 100 pA), series resistance compensation was not usually used. Series resistances yielded after whole-cell access were typically less than 50 MΩ, giving rise to an error in command potential less than 5 mV for a 100 pA current.

High-resistance seals and whole cell access were obtained in fibers constantly perifused with the extracellular recording solution. Control fibers remained under the perifusion for the length of the trial. In test groups after the inward current reached a steady state (1.5 min after the start of the trial), the lines supplying perifusion system were switched from control to drug-containing bathing solution for 2.5 minutes followed by washout with control solution.

For all of the pharmacology experiments on the inward currents, the fibers were voltage-clamped at V_h_ of −70 mV and depolarized to +20 mV for 200 ms twice every 30 sec. Leak and capacity currents were assessed and subtracted online from test currents using a P/2 protocol in pCLAMP software. All subsequent data analysis employed averaged leak subtracted current values.

Peak current values used corresponded to peak amplitudes within first 50 ms of the 200 ms test pulse by 5 point smoothing. The sustained current values corresponded to mean amplitudes within the last 50 ms of the 200 ms test pulse. For the plotting of graphs, individual peak and sustained leak subtracted values from each fiber were first normalized with respect to the peak and sustained current values at 1.5 min after the start of the trial for this particular fiber. Then graphs showing baseline current amplitude change during application and after washout of drugs were made from the normalized data.

## Results

### FLP-Induced Contractions Require Extracellular Ca^2+^

**Figure 2:**
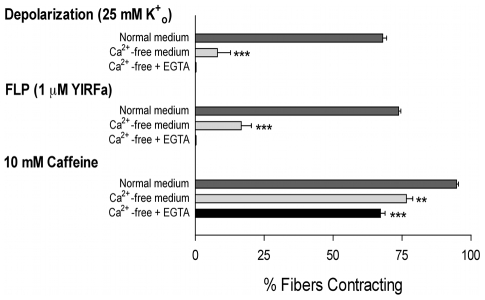
The FMRFamide-like peptide (FLP)-induced contractions are dependent on the presence of extracellular Ca^2+^. In normal extracellular medium containing 4.1 mM Ca^2+^, the muscle fibers contract in response to exposure to the flatworm FLP YIRFamide, depolarization with elevated extracellular K^+^ or caffeine. If the extracellular Ca^2+^ is omitted, the FLP- and the depolarization-induced contractions are significantly diminished; if 0.5 mM EGTA is included, both are completely blocked. In contrast, caffeine-induced contractions are not nearly as affected by the exclusion of extracellular Ca^2+^. Each data point is the mean ± SEM of % muscle fibers contracting, with n≥5 for each point. Each treatment is compared to control, **P<0.01, ***P<0.005.

We have previously reported that flatworm FMRFamide-like peptides (FLPs) induce concentration-dependent contraction of individual, dispersed schistosome muscle fibers, and that contracted fibers relax with the cessation of FLP application [9], [10] (Figure 1). In order to investigate the source of the Ca^2+^ utilized in the FLP-induced contractions, we attempted to induce those contractions in varied extracellular Ca^2+^ conditions. In the contraction assay, the individual muscle fibers are bathing in a modified Dulbecco's Modified Eagle Medium (DMEM) that contains 4.1 mM Ca^2+^; the fibers are then perifused with the test compounds dissolved in the same modified DMEM. When Ca^2+^ is simply omitted from those media producing nominally Ca^2+^-free media, the percentage of muscle fibers contracting in response to FLPs drops from 74±2% to 15±4%, and when 10 µM EGTA is added, FLP-induced contractions are completely abolished (Figure 2).

These fibers do contain sufficient intracellular stores of Ca^2+^ to support contraction. For example, under these same conditions in which extracellular Ca^2+^ is taken away, caffeine can elicit contraction via a ryanodine-sensitive intracellular store [18]. Since YIRFamide does not elicit contractions under the same conditions, it demonstrates that the FLP contraction does not utilize this intracellular store, and reinforces the conclusion that the FLP-induced contractions depend on an influx of extracellular Ca^2+^ (Figure 2). Together, these results show that the FLP-induced contractions require extracellular Ca^2+^, even though there are stores of intracellular Ca^2+^ sufficient to support contraction.

### FLP-Induced Contractions Utilize Voltage-Operated Calcium Channels

Next, we explored the conduit of Ca^2+^ entry. We have previously reported that depolarization-induced contractions of these same fibers are dependent on the presence of extracellular Ca^2+^ and that those contractions use voltage-operated Ca^2+^ channels (VOCCs) [19], because some classical VOCC inhibitors can block them. So, our first hypothesis was that the FLP-induced contractions were also employing VOCCs present in the sarcolemma. To test that hypothesis, we tested the ability of a panel of VOCC blockers to block the FLP-induced contractions.

**Figure 3:**
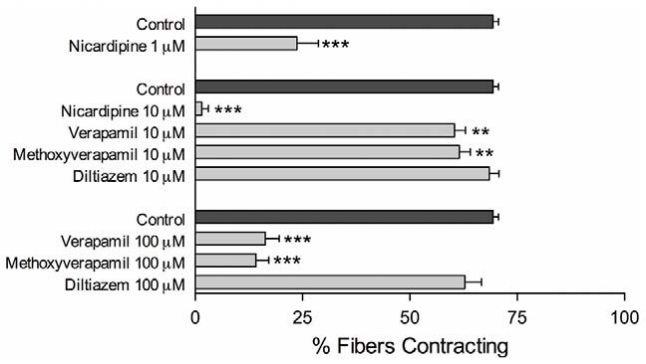
The FMRFamide-like peptide (FLP)-induced contractions are sensitive to classical voltage-operated Ca^2+^ channel (VOCC) blockers. Although it required relatively high concentrations, the FLP-induced contractions were significantly blocked by the dihydropyridine nicardipine, as well as the phenylalkylamines verapamil and methoxyverpamil, but not by the benzothiazipine diltiazem. The potency and the rank order of efficacy of these VOCC blockers is the same as have been reported previously for the blockade of depolarization-induced contractions: nicardipine > verapamil ≈ methoxyverapamil >>> diltiazem. The drugs were added 15 min before testing with 1 µM YIRFamide. Data points represent the mean ± SEM of % muscle fibers contracting, with n≥5 for each point. **P<0.01, ***P<0.005.

The FLP-induced contractions are blocked by relatively high concentrations of some VOCC inhibitors, just as are the depolarization-induced contractions (Figure 3). The dihydropyridine nicardipine is the most potent blocker of the YIRFamide-induced contractions, and it was previously shown to be the most potent blocker of depolarization-induced contractions of the same fibers [19]. Verapamil and methoxyverapamil (D-600) are both effective blockers of the FLP-induced contractions, but they require relatively high concentrations; control contractions were reduced from 72±2% to 16±3% in 100 µM verapamil, and 14±3% in 100 µM methoxyverapamil. The benzothiazipine diltiazem was ineffective even at 100 µM; it was likewise quite ineffective at blocking the depolarization-induced contractions at this concentration.

These pharmacological data are somewhat equivocal in that fairly high concentrations of the VOCC blockers are required for inhibition of the contractions. This is not uncommon when applying vertebrate pharmacological tools to these very early-diverging invertebrates. For example, in two different jellyfish preparations, very high concentrations of dihydropyridines and phenyalkylamines were needed to block VOCCs [20], [21]. However, the similarity of the rank order and potency of various blockers on contractions elicited by depolarization and those elicited by FLPs suggest that the pathway of Ca^2+^ entry for both are the same, supporting the hypothesis and implicating VOCCs in FLP-induced Ca^2+^ entry.

### Voltage-Operated Ca^2+^ Currents in Schistosome Muscle

Our next hypothesis was that the FLPs are in some way activating or enhancing the VOCCs in the sarcolemma. In order to test this hypothesis, we first had to develop an approach to measuring the VOCC currents of schistosome muscle fibers and their modulation by exogenous modulators (such as VOCC blockers or FLPs). Although we have measured VOCC currents in these fibers previously using whole-cell patch-clamp techniques [19], pharmacological assessments have been difficult, as the currents are quite small and it is hard to distinguish a pharmacological effect from the somewhat variable but very rapid rundown.

#### Amplifying the inward currents by providing additional charge carrier

In order to better resolve the inward currents, we tested a number of different extracellular bathing media. In the standard extracellular bathing medium which contained 2.0 mM Ca^2+^, inward currents were detectable, but usually too small to lend themselves to ongoing study. Increasing the extracellular Ca^2+^ as high as 10 mM amplified the inward currents, but also dramatically changed the appearance of the fibers, causing a high percentage of them to contract which made it difficult to find a sufficient number of relaxed fibers from which to record. When the extracellular bathing medium was supplemented with Ba^2+^, the currents were also amplified, but the fibers did not contract. The inward currents were most consistently recorded when the 2.0 mM Ca^2+^ normally present in the extracellular medium was supplemented with 15 mM Ba^2+^. This proved successful in enhancing the amount of available charge carrier without producing dramatic untoward effects on the isolated muscle fibers.

#### A protocol for managing rapid rundown

**Figure 4:**
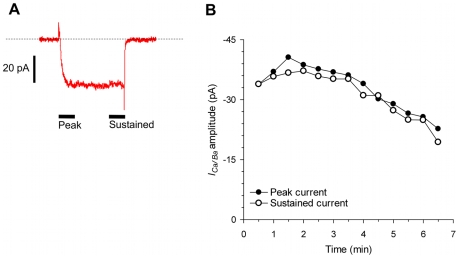
Voltage-gated inward currents occur in schistosome muscle fibers, but they rapidly rundown. (A) An inward current is evoked by a 200 ms depolarizing step to +20 mV from a holding potential of -70 mV in the presence of 2 mM extracellular Ca^2+^ and 15 mM extracellular Ba^2+^. The dashed line represents the baseline current level, the horizontal bars show the times from which the peak and sustained current values were derived, and the vertical scale bar is 20 pA. (B) The inward current runs down with time. On average, currents reached peak amplitude 1.5 minutes after the start of the trial and maintained an approximate steady level for the next 3-6 min followed by steady decline in amplitude. This graph shows that peak and sustained current amplitudes for the same muscle fiber depicted in (A).

We found that the inward currents of these contractile schistosome muscle fibers run down rapidly (Figure 4), as we have noted earlier [19] and as has been reported for a number of VOCC currents in nerve and muscle from other animals [22], [23], [24], [25]. After we obtained whole-cell access by disrupting the membrane under the pipet via suction, the first minute was characterized by an increase in the inward current, presumably attributable to reaching some equilibrium between the intracellular solution in the pipet and the contents of the cell. After seeing a peak inward current somewhere near 1.5 min, there was a relatively stable current for the next 2 to 3 min, followed by a steady decline in current amplitude. For most muscle fibers, the inward current was less than 20% of maximum within 8 min of whole-cell access.

Therefore, the window in which we could measure exogenously-induced increases or decreases in current was, at best, limited to a time period between 1.5 min and 8 min after whole-cell access; better was to restrict pharmacological examination to between 1.5 min and 4.5 min. We settled on a protocol in which exogenous agents were perifused onto the voltage-clamped fibers between 2.0 and 4.5 min after whole-cell access, and experimental fibers were always compared to control-perifused fibers in order to further control for rundown.

### Pharmacology of Voltage-Operated Calcium Channels in Schistosome Muscle

**Figure 5:**
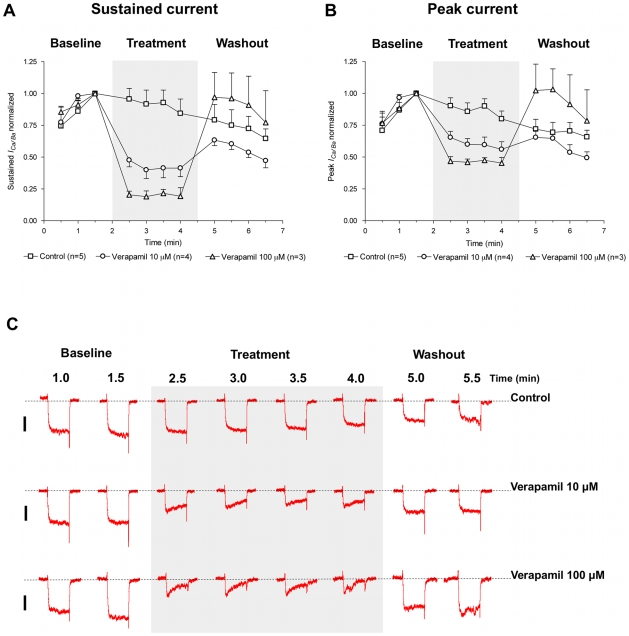
The phenylalkylamine voltage-operated Ca^2+^ channel blocker verapamil reversibly inhibits the inward current. Both the (A) sustained and (B) peak components of the inward current are significantly inhibited by 10 µM and 100 µM verapamil. For each fiber tested, in control and test groups, peak and sustained currents were normalized to corresponding current values obtained at 1.5 minute after the start of the trial. Data were plotted as mean ± SEM. Seals and break-in were performed with cells constantly perifused with control solution. Cells remained under the perifusion for the whole length of the trial. The shaded box indicates time points where cells were exposed to the drug-containing bathing solution (for the test groups) or control solution itself (for the control group); (C) Representative traces of *I_Ca/Ba_* activated by depolarizing *S. mansoni* muscle fibers from V_h_ of -70 mV to +20 mV with a 200 ms test pulse in the absence or presence of verapamil (10 µM or 100 µM). The dotted line denotes zero current level and the scale bar is 20 pA (traces from different fibers are scaled equally). The discontinuous time scale of the experiment is shown above traces, and each of the depolarizing pulses is 200 ms.

Both the peak and the sustained inward currents are inhibited by verapamil (Figure 5), the phenyalkylamine that also inhibited contractions elicited by FLPs and depolarization. Within 30 sec of application, 10 µM verapamil decreased the sustained current to 48±5% of maximum as compared to controls which showed no significant decrement over the same time (96±8%). At 100 µM verapamil the sustained current was decreased to 20±3%. The peak inward current was also inhibited by verapamil, but not as potently; the control peak current was 90±6% of the maximum, while it was decreased to 65±5% at 10 µM verapamil and 47±4% at 100 µM. Verpamil's inhibition was reversible.

Unfortunately, it was not possible to test the entire panel of VOCC blockers on the inward currents, because the dihydropyridines precipitated in the extracellular bathing medium containing high concentrations of divalent cations. Even at 10 µM, nicardipine and nifedipine both formed visible precipitate in the bathing medium with 15 mM Ba^2+^ and 2.0 mM Ca^2+^.

### Flatworm FLPs Enhance the Inward Currents in Schistosome Muscle

**Figure 6:**
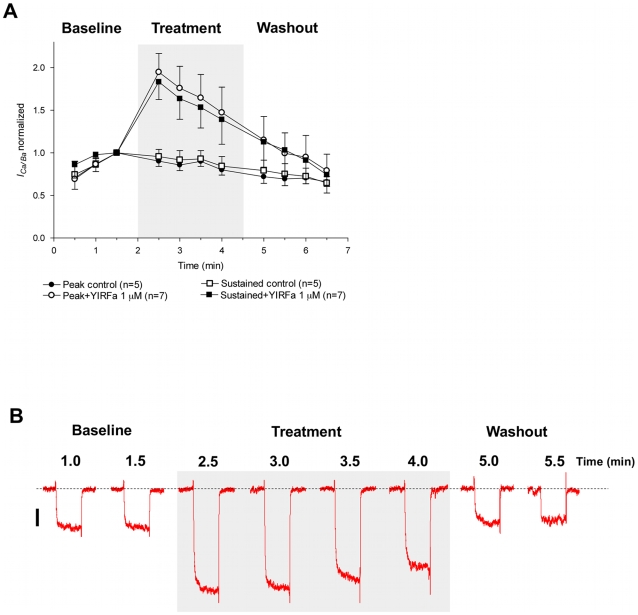
YIRFamide enhances whole cell voltage-gated *I_Ca/Ba_* in isolated *S. mansoni* muscle fibers. (A) Both peak and sustained *I_Ca/Ba_* increased in amplitude upon treatment with 1 µM YIRFamide. YIRFamide was applied as indicated with the shaded box. Data were normalized as described in Figure 4 and plotted as mean ± SEM. (B) Representative traces of *I_Ca/Ba_* before, during and after application of the peptide show the enhancement of the current and the washout of the effect after the cessation of application. The dotted line denotes zero current level and the scale bar is 20 pA. The discontinuous time scale of the experiment is shown above traces, and each of the depolarizing pulses is 200 ms.

Using the same protocols, the flatworm FLP YIRFamide significantly potentiates the inward currents (Figure 6). Within 30 sec after perifusion of the fibers with bathing medium containing 1 µM YIRFamide, the sustained and peak inward currents were both significantly enhanced compared to those perifused with bathing medium alone. Whereas the control fibers had rundown to 90±6% of their maximum value, the inward currents of the YIRFamide perifused fibers were 180±21% (sustained) and 190±22% (peak) of their pre-treatment maximum at the same time point.

The current potentiation was both transient and reversible. The fibers were perifused with the FLP for 2.5 min, and the most dramatic increases in the inward current amplitude were always seen immediately after exposure to the peptide. Even during the YIRFamide perifusion, the potentiation decreased toward control values over time, although they remained greater throughout. When the perifusion was shifted back to extracellular recording medium alone, the currents quickly returned to control levels.

**Figure 7:**
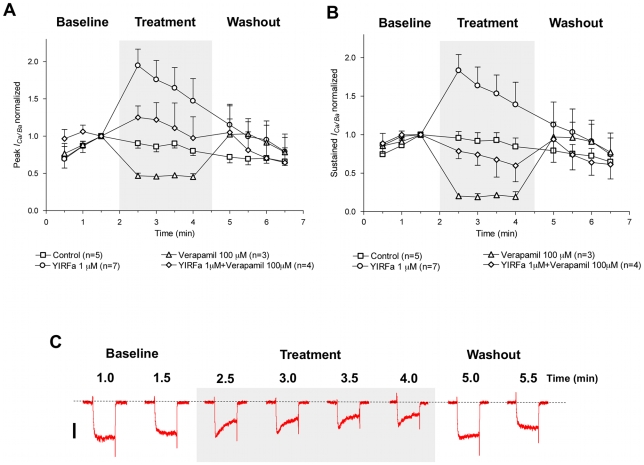
YIRFamide-mediated increase of *I_Ca/Ba_* in *S. mansoni* isolated muscle fibers is reduced during treatment with the Ca^2+^ channel blocker verapamil. Verapamil reduced the enhancement of *I_Ca/Ba_* produced by YIRFamide, for both the (A) sustained and (B) peak currents. In each graph, data are compared showing currents under control conditions, the inhibition produced by 100 µM verapamil, the enhancement produced by 1 µM YIRFamide, and the co-application of both verapamil and YIRFamide, which in both cases most closely resembles the control currents. (C) Representative traces of *I_Ca/Ba_* before, during and after treatment with 100 µM verapamil and 1 µM YIRFamide show currents that are effected by the co-application, but are intermediate to either agent applied alone. Data are plotted as mean ± SEM. Both verapamil and YIRFamide were dissolved in extracellular recording solution and delivered simultaneously during the time span indicated by a shaded box. The dotted line denotes zero current level and the scale bar is 20 pA. The discontinuous time scale of the experiment is shown above traces, and each of the depolarizing pulses is 200 ms.

In order to test the interaction between verapamil's inhibition and YIRFamide's stimulation of the VOCCs, we co-applied both simultaneously (Figure 7). The co-application resulted in VOCC currents that were neither as inhibited as verapamil treated currents, nor were they as enhanced as YIRFamide treated currents. With the co-application of 100 µM verapamil and 1 µM YIRFamide, the current most closely resembled control current.

### Examining the Biochemical Link Between YIRFamide and VOCCs

There is a striking parallel between the physiological data here and previous observations of protein kinase C (PKC)-mediated contractions of schistosome musculature, as induced by phorbol esters on whole male worms. PKC stimulation produces contraction of the muscle in a manner that depends on extracellular Ca^2+^ and is sensitive to nicardipine, leading the investigators to conclude that “activation of PKC in schistosomes with phorbol esters leads to muscle contracture by enhancing sarcolemmal Ca^2+^ channel activity” [26].

Other research suggests that most FLPs exert their effects through G protein-coupled receptors (GPCRs). Specifically germane to this model, a GPCR has been demonstrated to be a receptor for GYIRFamide in the flatworm *Girardia tigrina* [27]. Physiological data related to FLP contractions of flatworm muscle also implicate GPCRs in a number of species [7], [14], [17]. Working with *Fasciola hepatica*, Graham et al. found that GYIRFamide and other FLPs induced increased frequency and amplitude of muscle strip contractions [14]. They also found that these FLP-induced contractions involved G-protein mediated signaling and were mimicked by phorbol esters [17]. Chelerythrine chloride (10 µM) blocked the FLP-induced contractions of *Fasciola*, pointing to a PKC-dependent pathway.

Together, these data lead to a hypothesis that a PKC pathway is involved in FLP-induced contractions of schistosome muscle, and this hypothesis is granted further credibility because GPCRs are known to enhance VOCC currents via a PKC pathway in some other systems. For example, metabotropic glutamate receptors have been shown to stimulate VOCCs from the Ca_V_2 family (N- and R-type calcium channels) through a protein kinase C pathway that depends on an interaction with the calcium channel β sub-units [28].

**Figure 8:**
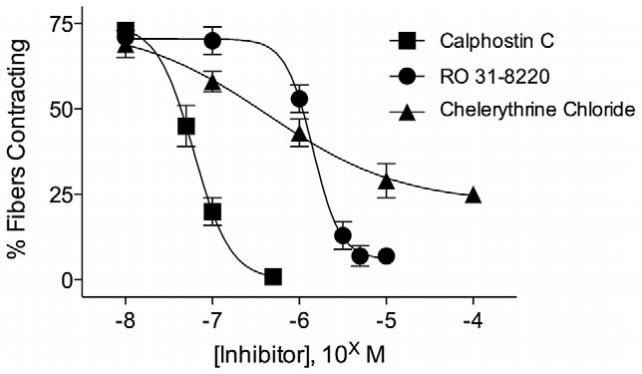
Protein kinase C (PKC) inhibitors block the FMRFamide-like peptide (FLP)-induced contractions. Concentration-response curves for the blockade of muscle fiber contractions by various PKC inhibitors show that the FLP-induced contractions use a PKC-dependent pathway. Both calphostin C (IC_50_ = 61 nM) and chelerythrine chloride (IC_50_ = 0.7 µM) block the contractions at concentrations well within the range attributed to specific PKC inhibition in other systems. RO 31–8220 also blocked the contractions, but at concentrations (IC_50_ = 1.54 µM) higher than are required for specific PKC inhibition in some mammalian systems. Data points represent mean ± SEM of % muscle fibers contracting, with n≥5 for each point.

We therefore tested a few PKC inhibitors to see if that pathway could be linked to the FLP-induced contractions. All of the PKC inhibitors we tested, calphostin C, RO 31–8220 and chelerythrine chloride, produced a concentration dependent block of contractions elicited by 1 µM YIRFamide (Figure 8). Calphostin C was the most potent, blocking with an IC_50_ = 61 nM, well within the concentrations at which it is considered specific for PKC inhibition [29]. Chelerythrine chloride specifically inhibits rat brain PKC at 0.7 µM, and also inhibited the neuropeptide contractions with an IC_50_ of 0.66 µM, well within the concentration typically considered specific for PKC inhibition in other systems [30]. The bisindolylmaleimide RO 31–8220 also blocked these contractions, but at relatively high concentrations (IC_50_ = 1.54 µM) which might be attributable to other, less specific effects of the compound.

## Discussion

Our goal was to elucidate the mechanism of FLP-induced contractions of *S. mansoni* muscle fibers. We found the FLP-induced contractions very much resemble depolarization-induced contractions both physiologically and pharmacologically. Specifically, the FLP-induced contractions utilize extracellular Ca^2+^ and the pharmacological profile implicates voltage-operated Ca^2+^ channels (VOCCs) as the conduit for entry into the muscle fibers. After developing a protocol to reliably measure inward VOCC currents and deal with their rapid rundown, we tested the effects of FLPs on these currents. We found that the VOCC currents were sensitive to the same blockers that inhibited the FLP-induced contractions. Then, we found that the application of FLPs enhance the inward VOCC currents, almost doubling their size.

Physiological data related to FLP contractions of flatworm muscle implicate GPCRs in a number of species [7], [14], [17]. There are a few well-documented cases of GPCRs linked to VOCC modulation, with many acting through a PKC pathway [28]. We found that the FLP-induced contractions in schistosome muscle are also dependent on a PKC pathway.

PKC has been implicated in muscle contraction in flatworms and even in neuropeptide-induced contractions. PKC activators (phorbol esters) have potent stimulatory effects on flatworm muscle, including schistosomes [17], [26]. Data here link these FLP-induced contractions to a PKC-dependent pathway and, therefore, the associated stimulation of VOCCs.

Neuronal VOCCs have been shown to be a target for enhancement by PKC [31], [32], [33], [34]. Heterologously expressed rat brain VOCCs can be stimulated by PKC activation, producing current increases of 130%–150% over controls. The PKC-mediated enhancement of the currents required co-expression of the non-conducting VOCC β subunits, and the PKC modulation involves a direct interaction with those β sub-units [28].

Interestingly, modulation of the interaction between schistosome calcium channel α and β subunits looks to be a target of the preeminent antischistosomal drug, praziquantel. Praziquantel interacts with structurally unusual schistosome VOCC β subunits to enhance current conducted through alpha subunits [35],[36].

This work elucidates some of the fundamental biology underlying schistosome neuromuscular biology. Specifically, that FLP neuromuscular excitation involves a PKC-dependent pathway leading to enhancement of Ca^2+^ influx through sarcolemmal VOCCs. Understanding the neuromuscular physiology of these worms is tantamount to understanding what has proven to be the most fertile field from which to harvest anti-schistosomal drugs.
